# Effects of a Reminiscence Therapy Program on Neuropsychiatric Symptoms and Quality of Life in People With Dementia: A Pilot Study Comparing Immersive Virtual Reality and Non-immersive Approaches

**DOI:** 10.1177/14713012251366348

**Published:** 2025-08-07

**Authors:** Maria Soares, Vanessa Quental, Miguel Pereira, Ana Isabel Corregidor Sánchez, Ana Costa, Paula Portugal, Tiago Coelho

**Affiliations:** 1 E2S, Polytechnic of Porto, Porto, Portugal; 2 Faculty of Health Sciences, University of Castilla la Mancha, Talavera de la Reina, Spain; 3 InveTES Research Group, University of Castilla la Mancha, Spain; 4 Santa Casa da Misericórdia de Vila Nova de Gaia, Vila Nova de Gaia, Portugal; 5 LabRP/CIR, E2S, Polytechnic of Porto, Porto, Portugal

**Keywords:** dementia, virtual reality, reminiscence therapy, behavioral and psychological symptoms of dementia, quality of life

## Abstract

This study compared the impact of an immersive virtual reality (VR)-based reminiscence therapy program with a similar non-immersive intervention on neuropsychiatric symptoms and quality of life of people with dementia. A pilot randomized controlled trial was conducted with 14 individuals with mild to moderately severe dementia, who participated in eight biweekly individual reminiscence sessions conducted by trained researchers, in which 360° videos of locations with personal relevance were displayed. Participants were randomly divided in two groups: one receiving therapy using VR headsets to promote an immersive experience while the other watched the videos on a monitor (non-immersive approach). Assessment was conducted pre- and post-intervention using the Quality of Life in Alzheimer’s Disease Scale to measure quality of life and the Geriatric Depression Scale, Generalized Anxiety Disorder Scale and Neuropsychiatric Inventory to evaluate neuropsychiatric symptoms. Adverse simulation-related symptoms were also assessed with the Simulator Sickness Questionnaire. There were no statistically significant differences between groups at baseline, regarding sociodemographic variables and level of dementia progression. The results indicated significant improvements post-intervention in quality of life from caregivers’ perspectives in the non-immersive group (*p* < .05) but not in the VR group. Differences in overall neuropsychiatric symptoms, depressive symptoms and anxiety symptoms between assessments were non-significant. However, slight improvements were visible, particularly regarding anxiety. Only a few instances of mild cybersickness symptoms were reported in both groups. In this study, pre- and post-intervention comparisons do not support the added value of using immersive VR in reminiscence therapy for people living with dementia. This raises questions about whether VR is worthwhile compared to traditional approaches, and how to better capture potential benefits of immersion with VR, which may be more evident considering in-session engagement and well-being or longer intervention programs.

## Introduction

Dementia, which is usually caused by a progressive neurodegenerative disorder, is a syndrome characterized by cognitive decline and impaired daily functioning, which significantly diminishes the quality of life (QoL) of affected individuals and their caregivers (Livingston et al., 2020; World Health Organization, 2021). As the global population ages, the number of persons with dementia continues to rise, posing a major challenge to healthcare systems (Livingston et al., 2020). Alongside cognitive deficits, dementia is often accompanied by neuropsychiatric symptoms such as depression, anxiety, apathy, and agitation (Gottesman & Stern, 2019; Radue et al., 2019; Savva et al., 2009). These symptoms can exacerbate the limitations in cognitive and functional performance, increase caregiver burden, and lead to higher rates of institutionalization (Cipriani et al., 2020; Feast et al., 2016; Saari et al., 2020).

Non-pharmacological interventions are crucial in dementia care, as they contribute to manage symptoms and improve well-being without the side effects frequently associated with medication (Abraha et al., 2017; Dyer et al., 2018; Kishita et al., 2020; National Institute for Health and Care Excellence, 2018; Van De Glind et al., 2013; Zucchella et al., 2018). Among these interventions, reminiscence therapy is widely used and involves recalling past experiences, often facilitated by visual, auditory, or other sensory stimuli, to stimulate memory and emotional engagement (Cuevas et al., 2020; Huang et al., 2015; Park et al., 2019; Woods et al., 2018). This approach has shown potential benefits in reducing neuropsychiatric symptoms and improving cognitive function, communication, and QoL (Huang et al., 2015; Kishita et al., 2020; Park et al., 2019; Woods et al., 2018).

Advances in virtual reality (VR) technology have introduced new possibilities for enhancing reminiscence therapy by creating realistic and immersive environments that allow individuals to experience a sense of presence in significant or familiar settings (Coelho et al., 2020; Lu et al., 2023; Niki et al., 2021; Strong, 2020). While some studies suggest that VR-based reminiscence therapy may enhance engagement and evoke stronger emotional responses compared to traditional non-immersive approaches, the evidence remains limited and mixed, with some research indicating no significant differences in clinical outcomes (Kirk et al., 2019; Lu et al., 2023; Saredakis et al., 2020, 2021; Strong, 2020). Consequently, it is important to further investigate the added therapeutic value of immersive VR in reminiscence therapy.

This study aims to analyse the effects of an immersive VR-based reminiscence therapy program on neuropsychiatric symptoms and QoL in individuals with dementia, in comparison with a similar non-immersive approach.

## Methodology

### Study Design and Participants

A quantitative pilot randomized controlled trial, with assessments pre- and post-intervention, was conducted, with participants being randomly assigned to one of two groups (experimental: immersive VR-based reminiscence therapy; control: non-immersive reminiscence therapy) using a 1:1 allocation ratio. Participants were recruited in institutions that provided health and social services to older adults in the district of Porto, through a non-probabilistic sampling process. Inclusion criteria consisted of being aged 65 years or older and having a clinical diagnosis of dementia. Exclusion criteria were severe visual deficits (since it could interfere significantly with the visualization of the videos), inability to verbally communicate (as communication was required for selected measurements), classification as being in stages 1–3 of the Global Deterioration Scale (Leitão et al., 2008; Reisberg et al., 1982) (considered as pre-dementia stages), being in a late stage of dementia (evaluated as in the seventh stage of the Global Deterioration Scale, due to very severe cognitive deficits and associated functional limitations), diagnosis of Lewy body dementia (due to higher probability of developing hallucinations (D’Cunha et al., 2019), diagnosis of other psychiatric disorders (in order to reduce confounding effects), and not having family members or other relevant individuals who could provide information about the participants’ life story and complete the assessments. Recruitment resulted in 16 individuals being included in the study, with 8 participants being allocated to each group. The allocation of participants was conducted using a double-stratified randomization approach to ensure that the groups were comparable. The randomization was stratified by institution where the participant was recruited, as the activities and services provided by the different institutions varied. Additionally, randomization was also stratified based on the progression of dementia (based on the Global Deterioration Scale), to ensure that each group included participants at various stages of dementia severity. A researcher not involved in the assessments conducted the randomization to minimize potential selection bias (Bhide et al., 2018; Brocklehurst & Hoare, 2017). Two participants allocated to the experimental group abandoned the study after pre-intervention assessment (one due to health reasons while the other decided not to participate in the intervention), resulting in a final sample of 14 individuals. All participants signed informed consents which included the objective of the study, the assurance of confidentiality, and the right to withdraw at any time without any negative consequences (study number CE0024B, approved by the Ethics Committee of the School of Health – E2S, Polytechnic of Porto).

### Measurements

Participants were assessed at baseline regarding sociodemographic variables (age, sex, marital status, and education), disability in basic daily activities (obtained with the Barthel Index) (Araújo et al., 2007; Mahoney & Barthel, 1965), cognitive status (measured with the Montreal Cognitive Assessment) (Freitas et al., 2011; Nasreddine et al., 2005), and dementia stage (classified with the Global Deterioration Scale) (Leitão et al., 2008; Reisberg et al., 1982). Researchers gathered and confirmed sociodemographic and disability-related information from participants and their caregivers. The Montreal Cognitive Assessment was conducted directly with participants, while the Global Deterioration Scale was evaluated based on previous assessments and input from caregivers and professionals at the recruiting institutions. Pre- and post-intervention assessments included evaluating QoL of the participants, from the participants’ and caregivers’ perspective, using the Quality of Life in Alzheimer’s Disease Scale (QoL-AD) (Barrios et al., 2013; Logsdon et al., 1999), as well as measuring neuropsychiatric symptoms in general, with the Neuropsychiatric Inventory (Cummings, 1997; Ferreira et al., 2014), and depressive and anxiety symptoms in detail with the 15-item Geriatric Depression Scale (GDS-15) (Apóstolo et al., 2014; Sheikh & Yesavage, 1986) and the Generalized Anxiety Disorder Scale (GAD-7) (Sousa et al., 2015; Spitzer et al., 2006), respectively. Also, symptoms related with simulation (also known as cybersickness) were measured in each intervention session using the Simulator Sickness Questionnaire (Carvalho et al., 2011; Kennedy et al., 1993). These scales will be briefly described below.

The Barthel Index (Araújo et al., 2007; Mahoney & Barthel, 1965) is a measure of disability in basic daily activities such as mobility, bathing, and feeding, with scores ranging from 0 to 20, where higher scores reflect greater independence. The Montreal Cognitive Assessment (Freitas et al., 2011; Nasreddine et al., 2005) is a screening tool that assesses various cognitive domains, including mental functions such as memory, executive functions, language, and orientation, with a total score range of 0 to 30, where higher scores indicate better cognitive function. The Global Deterioration Scale (Leitão et al., 2008; Reisberg et al., 1982) is utilized to classify the stages of cognitive decline progression in dementia, along with associated behavioural and functional limitations, with scores ranging 1–7.

The QoL-AD (Barrios et al., 2013; Logsdon et al., 1999) evaluates the QoL in individuals with dementia, covering domains such as physical health, mood, memory, and social relationships. It includes 13 items rated on a 4-point scale (poor to excellent), providing a total score range of 13 to 52, resulting from the report of people with dementia and from their caregivers.

The Neuropsychiatric Inventory (Cummings, 1997; Ferreira et al., 2014) assesses the frequency (1-rarely to 4-very often) and severity (1- mild to 3-severe) of 12 neuropsychiatric symptoms commonly found in dementia: delusions, hallucinations, agitation, depression, anxiety, euphoria, apathy, disinhibition, irritability, aberrant motor activity, nighttime behavioural disturbances, and appetite changes. The scores for frequency and severity of each symptom are then multiplied, with the total score resulting from the sum of all scores (ranging from 0 to 144, considering that 0 is considered when the symptom is absent). It is administered to caregivers, who report on the recent behaviours of the person with dementia.

The GDS-15 (Apóstolo et al., 2014; Sheikh & Yesavage, 1986) and GAD-7 (Sousa et al., 2015; Spitzer et al., 2006) are self-report questionnaires assessing recent depressive and anxiety symptoms, respectively. The GDS-15 scores range from 0 to 15, while the GAD-7 scores range from 0 to 21, with higher scores on both scales indicating a greater number of symptoms.

The Simulator Sickness Questionnaire is used to detect symptoms of cybersickness that may occur during simulation experiences. It consists of 16 items (general discomfort, fatigue, headache, eyestrain, difficulty focusing, increased salivation, sweating, nausea, difficulty concentrating, fullness of head, blurred vision, dizziness with eyes open, dizziness with eyes closed, vertigo, stomach awareness, and burping), scored on a scale from 0 (none) to 3 (severe) (Carvalho et al., 2011; Kennedy et al., 1993). In the present study, this measure was administered before and after each session.

### Intervention

The intervention consisted of eight individual reminiscence sessions, conducted twice a week for four weeks. Sessions lasted approximately 20 minutes and took place in a quiet room with a trained researcher (occupational therapist) guiding the reminiscence process. Oculus Quest VR headsets were used to present 360° videos to participants in the experimental group while those in the control group watched videos on a computer monitor. The content of the videos was personalized based on the participants’ life history, gathered through preliminary interviews with family members or other relevant individuals. Videos consisted of locations with personal relevance, filmed with Action Cam 360 GoPro Fusion cameras or selected from a multimedia library created in result of previous research (Coelho et al., 2020). This individualized approach aimed to maximize engagement and evoke positive memories during the sessions. The procedure included a structured script for each session to standardize the interaction across groups, with prompts encouraging participants to describe their experiences and memories related to the content. Participants were free to explore the environments actively, either by head movements in the immersive intervention or by moving the computer mouse in the non-immersive intervention. In the latter case, if participants preferred, they could request the researcher to operate the computer mouse on their behalf. During the immersive VR sessions, the content viewed by participants through the headsets was simultaneously displayed on an external computer monitor, allowing the therapist to follow the participants’ exploration of the environment in real time and support a contextualized discussion throughout the session. Researchers were also instructed to interrupt the intervention if any severe neuropsychiatric or cybersickness symptom was expressed during the session.

### Analysis

Data were analysed using SPSS software (version 27.0). Quantitative data were described using proportions, mean values, and standard deviations, according to the nature of the variables. Baseline characteristics were compared between groups using independent samples t-tests or Mann-Whitney tests for continuous variables, and Fisher’s exact tests for categorical variables. Differences between assessments within each group were assessed using Wilcoxon signed-rank tests. Statistical significance was set at *p* < .05.

## Results

The mean age of the participants was approximately 85 years, with most being women and widowed (64.3%). Mean education was approximately 6 years. Regarding institution where participants were recruited, 7 (50%) were from a nursing home and 7 (50%) were clients of a day-care centre. The mean Barthel Index score was 15.1 and regarding cognitive status, the mean Montreal Cognitive Assessment score was 8.2 and most participants (85.7%) presented a moderately severe cognitive decline, measured by the Global Deterioration Scale. There were no statistically significant differences between groups regarding these variables (Table 1).

**Table 1.** Participant Characteristics at Baseline (Sociodemographic Variables, Institution Where Participants Were Recruited, Disability and Cognitive Status) and Comparison Between Intervention Groups

	Sample (n = 14)	Immersive VR intervention group (*n* = 6)	Non-immersive intervention group (*n* = 8)	
	*n* (%)	*n* (%)	*n* (%)	*p*-value
Age (years), mean±SD	85.50 ± 7.87	84.67 ± 3.24	86.13 ± 2.94	.75^a^
Sex				
Women	9 (64.3)	3 (50)	6 (75)	.58^b^
Men	5 (35.7)	3 (50)	2 (25)	
Marital status				
Married	5 (35.7)	2 (33.3)	3 (37.5)	1.00^b^
Widowed	9 (64.3)	4 (66.7)	5 (62.5)	
Education (completed school years), mean±SD	6.21 ± 4.59	7.50 ± 4.93	5.25 ± 4.40	.47^c^
Institution where participants were recruited				
A (nursing home)	7 (50)	2 (33.3)	5 (62.5)	.59^b^
B (day-care centre)	7 (50)	4 (66.7)	3 (37.5)	
Barthel index score (0–20), mean±SD	15.14 ± 4.19	17.17 ± 2.32	13.63 ± 4.75	.22^c^
Montreal Cognitive assessment score (0–30), Mean±SD	8.21 ± 3.56	9.67 ± 3.62	7.13 ± 3.31	.33^c^
Global deterioration scale				
Moderate cognitive decline (stage 4)	1 (7.1)	---	1 (12.5)	1.00^b^
Moderately severe cognitive decline (stage 5)	12 (85.7)	6 (100)	6 (75)	
Severe cognitive decline (stage 6)	1 (7.1)	---	1 (12.5)	

^a^Independent samples t-test.

^b^Fisher’s exact test.

^c^Mann-Whitney test; Virtual Reality (VR).

Regarding adverse simulation-related symptoms assessed with the Simulator Sickness Questionnaire, there were very few instances of symptoms being reported or increases in symptom severity after the sessions. For most symptoms, only 1 or 2 total cases of slight worsening were reported across the 8 sessions for both groups. Additionally, increases in cybersickness were more common in the control (non-immersive) group than in the experimental (immersive) group. Symptom exacerbation was more frequently reported for difficulty concentrating (8 cases in the control group vs. 5 cases in the experimental group), difficulty focusing (6 cases in the experimental group vs. 5 cases in the control group), and blurred vision (6 cases in the control group vs. 2 cases in the experimental group). No severe symptoms were recorded that required session termination.

Regarding QoL, overall neuropsychiatric symptoms, depressive and anxiety symptoms, statistically significant differences (*p* < .05) between pre- and post-intervention assessments were only found in the control group, with QoL as rated by the participants’ caregivers improving after intervention. Although QoL in caregivers’ perspective also increased in the immersive VR group, this difference was not statistically significant. Regarding self-rated QoL, there was a decrease after intervention in both groups, but this difference was not significant. Regarding the score of Neuropsychiatric Inventory for overall neuropsychiatric symptoms as described by caregivers, results were relatively similar in both assessments for both groups (particularly considering the possible score range of 0-144). Finally, depressive and anxiety symptoms declined from pre- to post-intervention in both groups, but these differences were also not statistically significant (Table 2).

**Table 2.** Comparison of QoL, Overall Neuropsychiatric Symptoms, Depressive and Anxiety Symptoms Between Pre- and Post-Intervention for Each Group

	Intervention group	Pre-intervention	Post-intervention	
		Mean ± SD	Mean ± SD	*p*-value
QoL-AD: Self-rated (13–52)	Immersive VR	34.00 ± 2.53	36.33 ± 4.27	0.17
	Non-immersive	34.63 ± 5.01	33.63 ± 2.88	0.50
QoL-AD: Rated by caregiver (13–52)	Immersive VR	31.00 ± 5.44	34.00 ± 8.30	0.23
	Non-immersive	32.25 ± 4.46	34.25 ± 5.01	**0.048**
Neuropsychiatric inventory (0–144)	Immersive VR	13.00 ± 9.07	13.50 ± 12.03	0.67
	Non-immersive	16.75 ± 16.36	17.88 ± 25.83	0.89
GDS-15 (0–15)	Immersive VR	5.00 ± 3.52	3.17 ± 2.99	0.10
	Non-immersive	6.12 ± 3.94	5.13 ± 3.23	0.57
GAD-7 (0–21)	Immersive VR	6.33 ± 5.54	2.83 ± 3.66	0.07
	Non-immersive	6.75 ± 7.59	2.13 ± 2.64	0.06

Virtual Reality (VR); Quality of Life in Alzheimer’s Disease Scale (QoL-AD); Geriatric Depression Scale (GDS-15); Generalized Anxiety Disorder Scale (GAD-7). Bold indicates p-values below 0.05.

## Discussion

This study evaluated the effects of an immersive VR-based reminiscence therapy compared to a non-immersive approach, focusing on neuropsychiatric symptoms and QoL of individuals diagnosed with dementia. The findings revealed statistically significant improvements only in regard to caregiver-rated QoL in the non-immersive intervention group. Indeed, for the other variables examined in the present study, including neuropsychiatric symptoms and self-rated QoL, no significant differences were found between pre- and post-intervention assessments in either group. While reductions in depressive and anxiety symptoms were observed in both groups, these changes did not reach statistical significance.

Although reminiscence therapy has been linked to improvements in QoL and a reduction in neuropsychiatric symptoms in dementia (Huang et al., 2015; Kishita et al., 2020; Park et al., 2019; Saragih et al., 2022; Woods et al., 2018), the benefits observed in this study were limited. While there were some positive changes in scores, they did not reach statistical significance for most variables. This may be attributed to several factors, such as the short intervention period of just four weeks, which may have been too brief to achieve clinically meaningful changes, even though the study included eight sessions, meeting the minimum recommended number of sessions for a reminiscence program (Cuevas et al., 2020; Macleod et al., 2021; Saragih et al., 2022; Woods et al., 2018). Additionally, the duration of each session (approximately 20 minutes) may not have been sufficient to produce notable symptom improvements, as individual reminiscence therapy sessions with people with dementia are usually longer (30–60 minutes) (Cuevas et al., 2020; Macleod et al., 2021; Saragih et al., 2022; Woods et al., 2018) However, there is evidence that exposure to immersive VR for up to 20 minutes is generally well-tolerated, and longer exposure may increase the risk of cybersickness and overall discomfort, associated not only with immersion in the virtual environment but also with the weight and pressure exerted by the VR headset (Appel et al., 2024; Lu et al., 2023; Saredakis et al., 2021).

Another important consideration is the heterogeneity of dementia and the varying ways in which participants respond to this type of intervention (Kishita et al., 2020; Livingston et al., 2020). With a small sample size, evident in the present study, these individual differences may have had a stronger impact on the outcomes, increasing the likelihood that the results would not reach statistical significance. Also, the limited number of participants may have amplified the effect of negative memories surfacing during the intervention, potentially restricting the measurable benefits post-intervention (Woods et al., 2018). A larger sample could have helped to mitigate these effects, providing greater statistical power to detect meaningful changes.

The lack of significant differences in the immersive VR group, compared to the improvements observed in the non-immersive group, warrants discussion, especially considering evidence suggesting that immersiveness enhances engagement and motivation, even if this does not consistently translate into improved therapeutic outcomes. (Lu et al., 2023; Saredakis et al., 2021; Strong, 2020). It is possible that although VR can increase emotional engagement and facilitate the exploration of the environment, as participants did not need to manipulate a mouse or request assistance to navigate, this did not translate into measurable clinical outcomes within the timeframe of the study. Moreover, while immersive environments can provide a more engaging experience, the therapeutic impact may still rely on other factors, such as the participants’ cognitive capacity to process and benefit from the simulated environments, which can vary widely in dementia (Kim et al., 2019; Niki et al., 2021). Additionally, in the non-immersive approach implemented in this study, the therapist was physically visible to the participants, which may have enhanced rapport and communication, potentially amplifying therapeutic outcomes. In contrast, the immersive VR experience, with headsets isolating participants visually, might have made the sessions feel more solitary, even though there was constant communication between the therapist and participant in both groups. This difference in perceived connection could have influenced the overall therapeutic experience.

Regarding cybersickness, very few symptoms were reported, and increases in symptom severity were minimal in both groups. As in previous studies (Appel et al., 2024; Coelho et al., 2020; Niki et al., 2021; Strong, 2020), the immersive VR intervention was well-tolerated, with no severe adverse effects that required session termination. The use of personalized content and structured guidance likely helped to prevent discomfort in both groups. However, slightly more symptoms were reported in the non-immersive group, potentially due to the need to use a computer mouse for navigating the environment or asking the researcher to perform this task. Additionally, viewing the video on a regular-sized computer monitor may have made visualization more challenging.

## Limitations

This study has several limitations. The small sample size limited the statistical power, reducing the ability to detect significant differences between groups. Moreover, no formal power calculation was conducted as participant recruitment was constrained by logistical factors, limiting the ability to estimate the appropriate sample size for detecting clinically meaningful effects. Additionally, mixed ANOVA tests could not be performed due to violations of statistical assumptions, potentially increasing the risk of Type I error in the analysis. Another limitation is the lack of blinding of both assessors and participants to the intervention groups, which may have introduced bias, particularly given the reliance on self-reported outcome measures. The length and duration of the intervention may also have been insufficient to capture the full effects of reminiscence therapy on the assessed variables. Some filmed locations were different from how participants remembered them from years ago, which could have hindered the evocation of related memories. Information on participants’ concurrent pharmacological and non-pharmacological treatments was not collected, which may have influenced the outcomes of the study. Furthermore, the selected measures may not have been sensitive enough to detect changes resulting from the intervention, as they might not fully capture the nuanced effects of reminiscence therapy.

Observational and qualitative data were not included, which could have provided additional insights into the added value of immersive VR experiences. Data collected through observation could have provided empirical evidence of participants’ engagement and emotional responses during the sessions, thereby illustrating the immediate impact of the intervention on their well-being. Conversely, qualitative methods could have been instrumental in exploring participants’ perspectives more deeply, possibly helping to contextualize individual responses to the intervention and shedding light on personal meaning, the biographical relevance of the content, and the nature of interaction during the sessions. Furthermore, qualitative approaches could have been employed to better understand caregivers’ perspectives regarding the impact of the intervention, which would be particularly important considering that the participants’ cognitive impairment could limit the depth and reliability of self-reports.

## Conclusion

This study demonstrated that immersive VR-based reminiscence therapy did not significantly outperform the non-immersive approach in improving neuropsychiatric symptoms or QoL for individuals with dementia. Indeed, the only statistically significant improvement was observed in the non-immersive group, where caregiver-rated QoL showed enhancement after the intervention, although the clinical relevance of this change is unclear. While both groups experienced reductions in depressive and anxiety symptoms, these changes did not reach statistical significance.

The findings have important implications for dementia care practice, suggesting that while immersive VR-based interventions offer innovative therapeutic opportunities, their benefits may not exceed traditional reminiscence methods in a short-term intervention context. Although immersive VR can enhance engagement and provide a more stimulating environment, the traditional non-immersive approach may have advantages, potentially due to the more direct and natural interaction with the therapist. This interpersonal element may foster a stronger therapeutic rapport and contribute to more effective outcomes. These results highlight the need for careful consideration of how immersive technology is integrated into dementia care, especially given the variability in cognitive functioning and behavioural and psychological symptoms among individuals with dementia.

Future research should explore the long-term effects of immersive VR reminiscence therapy, including larger sample sizes to increase statistical power and detect clinically meaningful changes. Given that the duration of each session must be limited to avoid discomfort and cybersickness, it may be particularly important to increase the total number of sessions to ensure adequate therapeutic dosage. Studies incorporating qualitative methods could also provide a deeper understanding of participants’ subjective experiences, which are not captured through quantitative measures alone. Additionally, incorporating observational data, rather than relying solely on self-reported outcomes, could offer a more accurate assessment of the effects of immersive VR on engagement and well-being during sessions. Furthermore, examining the influence of cognitive function and the therapeutic setting on intervention outcomes could help optimize the use of VR in dementia care. Continued investigation into innovative therapeutic approaches remains important for improving the QoL and symptom management in dementia.

## Supplemental Material

Supplemental Material - Effects of a Reminiscence Therapy Program on Neuropsychiatric Symptoms and Quality of Life in People With Dementia: A Pilot Study Comparing Immersive Virtual Reality and Non-immersive Approaches Supplemental Material for Effects of a Reminiscence Therapy Program on Neuropsychiatric Symptoms and Quality of Life in People With Dementia: A Pilot Study Comparing Immersive Virtual Reality and Non-immersive Approaches by Maria Soares, Vanessa Quental, Miguel Pereira, Ana Isabel Corregidor Sánchez, Ana Costa, Paula Portugal, Tiago Coelho in Dementia
