# Supplementary material for: Effects of a Reminiscence Therapy Program on Neuropsychiatric Symptoms and Quality of Life in People With Dementia: A Pilot Study Comparing Immersive Virtual Reality and Non-immersive Approaches
Source: Dementia (London). 2025 Aug 7;25(4):736–49. doi: 10.1177/14713012251366348 (PMC13062448; doi:10.1177/14713012251366348)
Supplement: Supplemental Material - Effects of a Reminiscence Therapy Program on Neuropsychiatric Symptoms and Quality of Life in People With Dementia: A Pilot Study Comparing Immersive Virtual Reality and Non-immersive Approaches [file sj-pdf-1-dem-10.1177_14713012251366348.pdf]

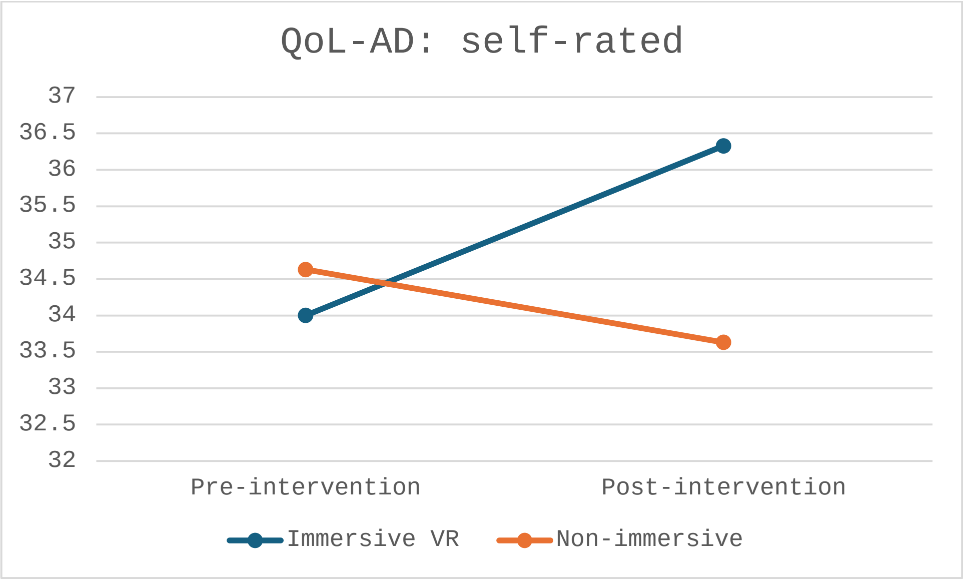

Figure 1: Mean scores of QoL-AD (self-rated) for pre- and post-intervention, by intervention group

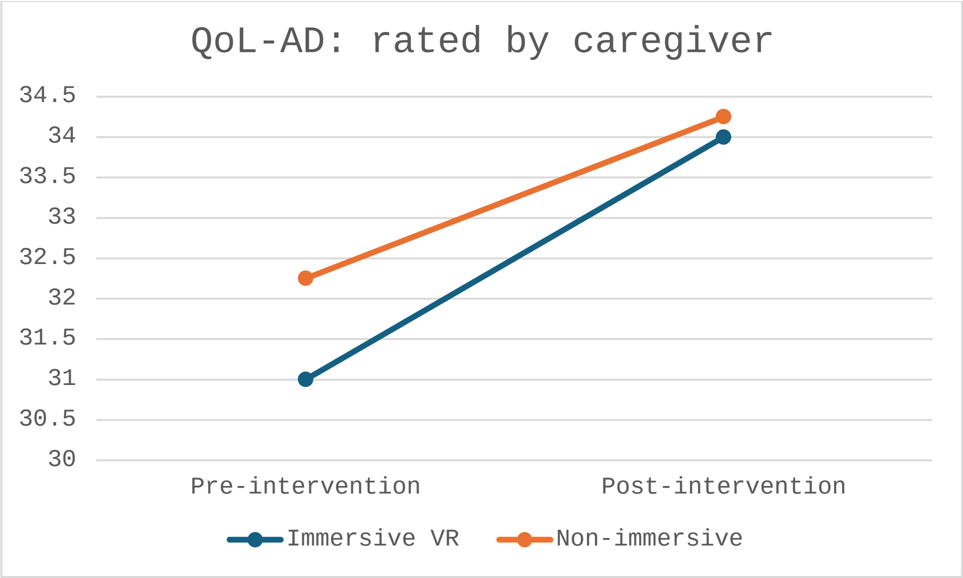

Figure 2: Mean scores of QoL-AD (rated by caregiver) for pre- and post-intervention, by intervention group

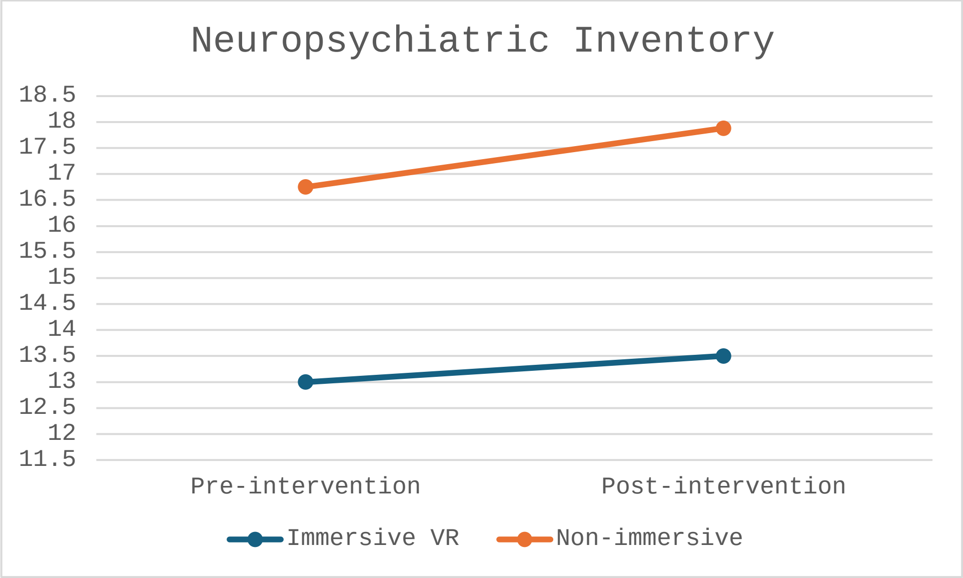

Figure 3: Mean scores of Neuropsychiatric Inventory for pre- and post-intervention, by intervention group

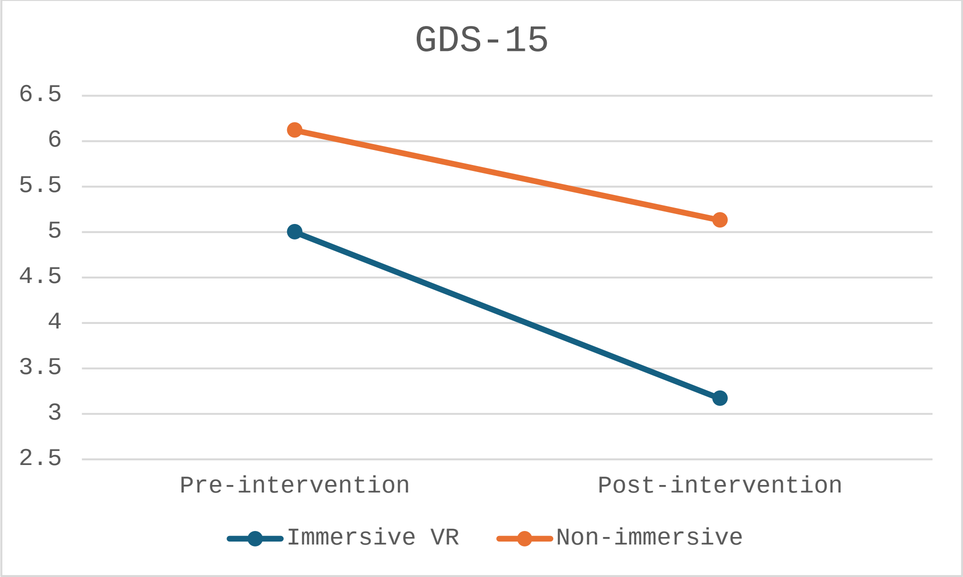

Figure 4: Mean scores of GDS-15 for pre- and post-intervention, by intervention group

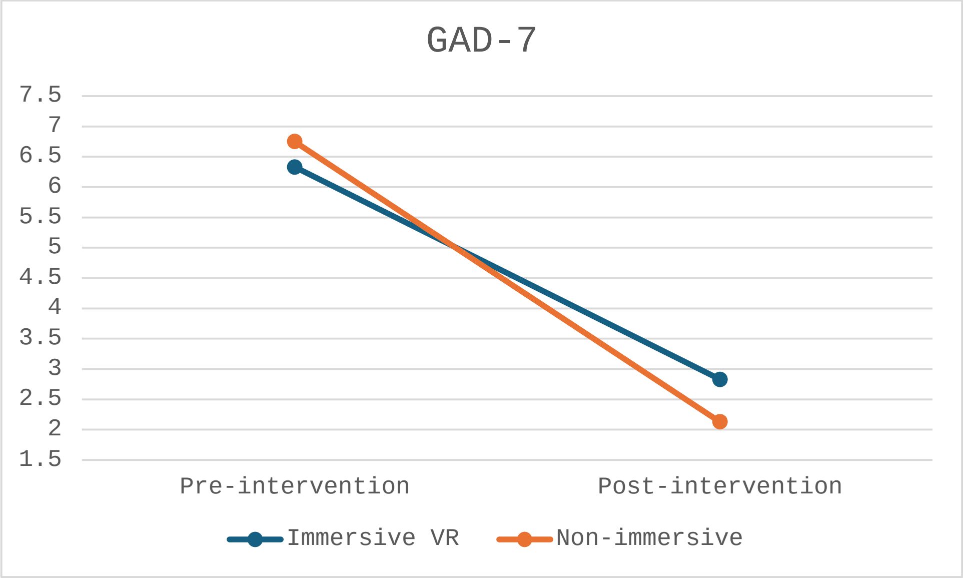

Figure 5: Mean scores of GAD-7 for pre- and post-intervention, by intervention group
